# Association between language and hearing disorders – risk identification

**DOI:** 10.6061/clinics/2017(04)04

**Published:** 2017-04

**Authors:** Alessandra Giannella Samelli, Silmara Rondon-Melo, Camila Maia Rabelo, Daniela Regina Molini-Avejonas

**Affiliations:** Disciplina de Fonoaudiologia, Departamento de Fisioterapia, Fonoaudiologia e Terapia Ocupacional, Faculdade de Medicina (FMUSP), Universidade de Sao Paulo, Sao Paulo, SP, BR

**Keywords:** Language Disorders, Hearing Disorders, Risk Assessment

## Abstract

**OBJECTIVE:**

To identify children at risk for hearing and/or language disorders and to investigate the association between these risks by conducting pre-validated hearing and language screenings.

**METHODS:**

The study was conducted during a polio vaccination campaign in August of 2013 in basic health units in western São Paulo. Parents of children between 2 and 5 years of age were asked to complete two screening tools: a hearing questionnaire (regarding hearing development) and a language production and comprehension scale (including the major language development milestones). The screening tools were administered by different researchers. We compared the risk of having language disorders among children at risk for hearing loss *versus* children not at risk, as well as the attributable risk and odds ratios. Chi-squared tests and logistic regression analyses were used.

**RESULTS:**

The study included 479 children with a mean age of three and one-half years, of whom 26.9% were identified as at risk for deficits in language production, 8.6% were at risk for deficits in language comprehension and 14% were at risk for hearing disorders. The children at risk for hearing disorders were twice as likely as those not at risk to exhibit language production and comprehension deficits.

**CONCLUSION:**

The results of this study highlight the importance of establishing and adopting low-cost procedures such as screenings to identify children at risk of developing language and/or hearing disorders in early childhood.

## INTRODUCTION

The question of whether hearing disorders caused by conductive problems are associated with language disorders remains controversial 1-3. While some authors report that conductive hearing loss can negatively affect the development of speech and language 3-5, others have found the opposite 1,7-9.

This lack of consensus among researchers warrants further investigation. A study with 1524 children with primary language impairment (PLI) found that children who had an abnormal audiological profile (excluding children with sensorineural hearing loss) were 63% more likely to have PLI than children who had a normal audiological profile 5.

Schönweiler et al. 4 emphasize the need to identify children with and without hearing loss in studies assessing speech and language, since hearing loss has been shown to play an important role in speech and language impairments.

Early intervention could substantially improve the development of communication among children who are at risk for or who have already been diagnosed with speech, language and/or hearing disorders. However, these children still face many barriers, and they may experience difficulty accessing professionals and health services for this type of intervention 10. Consequently, the use of low-cost strategies such as screenings and/or questionnaires could facilitate access and early identification among populations at risk for these disorders, especially in developing countries 11-14.

In the present study, we sought to identify children at risk for hearing and language disorders and to investigate the association between these risks using previously validated screening tools for each of the areas.

## PATIENTS AND METHODS

This cross-sectional study was approved by the ethics committee of the School of Medicine at the University of São Paulo (FMUSP) (n° 778/08 and 072/11).

The study was conducted during a polio vaccination campaign in August of 2013 in basic health units (Unidades Básicas de Saúde) in western São Paulo. Parents of children between 2 and 5 years of age were invited to participate in the study while their children were waiting to be vaccinated. The parents who agreed to participate were asked to sign an informed consent form and to fill out a socioeconomic form 15. The parents then completed the screening described below and received information regarding language and hearing development as part of a campaign promoting speech and hearing health.

1. Hearing Screening – this questionnaire was designed to identify children at risk for hearing loss 14 and includes questions about health history (pre-, peri-, and post-natal), development, communication skills, and hearing complaints. For each item, a response indicating risk for hearing loss received a score of 1. The total scores on the questionnaire were classified into two categories: 0-3 points ("passed") and 4 points or more ("failed"). The sensitivity of the questionnaire is 97.67%, and the specificity is 48.53% 14.
2. Language production (LP) and language comprehension (LC) screening – the American Speech-Language Hearing Association (ASHA) “How does your child hear and talk?” 16 language development scale considers all the major developmental milestones for LP and LC for each age group in early childhood. Each negative answer (indicating a risk for language disorders) received a score of -1, and each positive answer received a score of +1. A score of 0 was assigned when the parent was unsure of the answer to an item. A positive total score indicated the child “passed” the screening, while a negative score indicated failure. A score of 0 indicated that the child should be followed for at least one year. The questionnaire used in this study was a validated Portuguese version of the American tool published by the ASHA, and the age group division was consistent with the age group division in the original 17. This instrument's sensitivity is 94.4%, and its specificity is 82.4% 18.

It is important to note that the two screening tests were administered by different interviewers, which ensured that the interviewers who administered the hearing screening were unaware of the child's score on the LP and LC screening, and vice versa. The researchers trained the interviewers to ensure uniformity in the administration of the questionnaires. The questionnaires were tested in a pilot study, but the data were not included in the results of the present study.

All the subjects who failed one or both screenings were referred to comprehensive speech-language and/or hearing assessments. Following those assessments, any subjects identified as having language disorders and/or hearing loss were referred to an intervention.

We calculated the risk of language disorders among children at risk for hearing loss (**Risk for exposed**) and children not at risk for hearing loss (**Risk for unexposed**). We also measured the proportion of cases attributed to the risk factor that could be avoided if the risk factor were eliminated entirely (**attributable risk**) as well as the **odds ratio**, which indicates the odds that a specific outcome will occur given a particular exposure compared to the odds of the outcome occurring in the absence of that exposure.

Descriptive statistics and hypothesis tests were employed in the data analysis. Chi-squared tests were used to compare two categories of data, and ANOVAs were used to compare the means of the data. A logistic regression model was used to test the effect of risk associated with each variable after adjusting for age. The level of significance was set at 5%.

## RESULTS

The study included 479 children, 242 boys (50.5%) and 237 girls (49.5%), with a mean age of three and one-half years (SD: 1.73 years).

The distribution of the participating children by socioeconomic status is presented in Table 1.

Of the 479 children included in the study, 129 (26.9%) failed the LP screening, 41 (8.6%) failed the LC screening, and 67 (14%) failed the hearing screening.

The rate of failure on the LP and LC screening was significantly higher among the children who failed the hearing screening (Table 2). Whereas the children at risk for hearing loss had a failure rate of 49.25% (CI: 37.65-60.94%) on the LP screening, the children with no risk of hearing loss had a failure rate of 23.3% (CI: 19.47-27.63%). The children at risk for hearing loss had a 19.4% (CI: 11.57-30.56%) failure rate on the LC screening, while those with no risk had a failure rate of 6.79% (CI: 4.71-9.67%).

There were no significant differences between genders in terms of risk for LP or LC deficits (Table 3), indicating that gender does not influence the risk of having such deficits.

The children at risk for LP or LC deficits were younger than the children with no risk, but the difference was significant only for LP (Table 4).

No significant differences were observed for gender; therefore, we used a logistic regression model to compare the responses for the LP screening with those for the hearing screening, controlling for the age variable (adjusted).

Based on the estimates for the odds ratios and probabilities obtained from the logistic regression model for the LP screening, we observed that when comparing children one year apart in age, the probability of observing LP deficits in the older child was 0.79 times that of observing LP deficits in the younger child (lower limit=0.69; upper limit=0.91; *p*<0.01). In other words, in one year, the chance of LP deficits decreases approximately 21%.

The chance of exhibiting LP deficits was 3.84 times higher among the children with hearing deficits than among those without such deficits (lower limit=2.21; upper limit=6.7, *p*<0.01). That is, the chance of LP deficits was 2.84 times greater in the children with hearing deficits than in those without.

Table 5 presents combinations of the variables age (2 to 5 years) and hearing (deficit or no deficit) and their respective probabilities for LP deficits as estimated by the model.

According to the estimates of the odds ratios and probabilities obtained from the logistic regression model for the LC variable, we observed that the probability of exhibiting LC deficits was 3.30 times higher among the children with hearing deficits (lower limit=1.61; upper limit=6.76; *p*<0.01) than among those without. That is, the chance of having LC deficits was 2.30 times greater among the children with hearing deficits than among those without such deficits.

The estimated probabilities of exhibiting LC deficits according to the hearing screening results are presented in Table 6.

## DISCUSSION

In the current study, the observed rate of failure on the language screening was 26.9%. No studies of the rate of failure on language screenings are available in the literature. However, previous studies reported speech-language disorder prevalence rates ranging from 5% to 19%, depending on the criteria used to define the disorders and the age of the participants 20. It is important to highlight that this variability is due to discrepancies in the definition of speech and language disorders, the severity and type of communication impairments included in the definition, the nature of the surveyed population and differences in the methodological procedures used across the studies 21,22.

Additionally, socioeconomic status should be considered 12,13,23-26, as this variable may explain the greater failure rates observed on the language tests in our study relative to previous studies. In the current research, most of the participants belonged to economic class E (the lowest economic class according to the Brazilian classification), and most of the parents had not completed primary education, which differs from many of the previous studies 13,23-26.

In our study, the children had a failure rate of 8.6% on the LC screening. There are no previous population studies investigating LC deficits in children between 0 and 5 years of age. Considering that LC precedes LP, the results of the present study highlight the importance of early assessment of LC to understanding a child's capacity for developing LP skills 13. Furthermore, this knowledge is important for the provision of primary care and early intervention.

Consistent with the prevalence rates reported by previous studies, 14% of the participants in the current study failed the hearing screening 5,27-30. It is important to note that the prevalence of hearing loss in children varies from 3.9% to 24.5% worldwide, and the prevalence of middle ear disorders ranges from 7.3% to 36.2% 31. These differences in prevalence rates are likely due to the use of different evaluation protocols and to different characteristics of the studied populations, as noted by Olusanya 31.

The failure rate for LP and LC was significantly higher among children who failed the hearing screening. Thus, our findings support previous results suggesting that hearing loss can have a negative influence on the development of language in terms of both production and comprehension 4,5. The literature describes that deficits in attention, decoding, comprehension, memory, processing and the effective use of auditory information are among the types of damage that can result from even mild hearing loss 32. The aforementioned skills are closely related to the acquisition and development of language 33.

We did not observe significant differences between genders in terms of deficits in LP or LC. These findings are consistent with those of Tomblin et al. 34, whose results suggest that although boys generally present higher prevalence rates for language disorders, the difference is not statistically significant for PLI. Likewise, Pereira, Befi-Lopes and Samelli 5 found no significant interaction between gender and PLI. However, the results of a systematic review 35 revealed a trend for a higher prevalence of language disorders among males.

The children who failed the LP screening in the current study were significantly younger, on average. There is individual variation in language development, and this variation is most evident in the expressive language of younger children: one 18-month-old baby may use short sentences while another may produce only isolated words 36. Over time, these differences diminish, and skill levels begin to level off at approximately three years of age 37.

Because we identified that age played a role in LP failure, we compared responses for the LP screening to those for the hearing screening while controlling for age. This analysis revealed that a one-year increase in age resulted in a 21% lower probability of failing the LP screening. This finding confirms the results of a previous study, in which approximately half of the children who exhibited delays during the first months of language development eventually caught up with their same-age peers in subsequent years 38.

Given that untreated language disorders persist at a significant rate, ranging between 40 and 60% 20, it is very important to use screening for early detection and diagnosis and to provide immediate intervention 10. Screening should be performed regardless of age given the importance of language for an individual's overall development.

Identifying possible hearing loss is critical among children with language disorders, since the relationship between these variables can complicate individual profiles 4. In the present study, children at risk for hearing loss had a 2.84 greater chance of developing LP deficits than children with no risk for hearing loss. Similarly, children at risk for hearing loss were 2.30 times more likely than those with no risk to develop LC deficits. As indicated by previous studies 3-6, our findings highlight the influence of hearing on the development of expressive and receptive language.

This study found that of the children at risk, the largest proportion were at risk for LP disorders, followed by hearing loss and LC disorders.

Additionally, children at risk for hearing loss were twice as likely to display LP deficits as children with no risk for hearing loss. The same pattern was observed for LC deficits. The results of this study highlight the importance of establishing and adopting low-cost procedures such as surveys and screening for the early identification of children at risk for language and hearing disorders. Finally, our findings emphasize the need for health professionals to evaluate hearing in children with speech and language impairments and speech and language in children with hearing impairments as early as possible.

## AUTHOR CONTRIBUTIONS

All the authors participated sufficiently in the conception and design of the work, in the data analysis and in writing the manuscript to take public responsibility for it.

## Figures and Tables

**Table 1:** Distribution of children by socioeconomic status.

Socioeconomic status	A1	12	2.50
	A2	24	5.01
	B1	57	11.89
	B2	51	10.64
	C1	113	23.59
	C2	117	24.42
	D	93	19.41
	E	12	2.50

**Table 2:** Absolute (and relative) frequencies of LP and LC responses according to hearing screening results.

	Language Production			
Hearing	Not failed	Failed	Total	*p*-value (Chi-squared)
**Not failed**	316 (77%)	96 (23%)	412 (100%)	<0.0001
**Failed**	34 (51%)	33 (49%)	67 (100%)	

	Language Comprehension			
Hearing	Not failed	Failed	Total	*p*-value(Chi-squared)
**Not failed**	384 (93%)	28 (7%)	412 (100%)	0.0006
**Failed**	54 (81%)	13 (19%)	67 (100%)	

**Table 3:** Absolute (and relative) frequencies of LP and LC responses by gender.

	Language Production			
Gender	Not failed	Failed	Total	*p*-value(Chi-squared)
**Female**	177 (75%)	60 (25%)	237 (100%)	0.430
**Male**	173 (71%)	69 (29%)	242 (100%)	

	Language Comprehension			
Gender	Not failed	Failed	Total	*p*-value(Chi-squared)
**Female**	212 (89%)	25 (11%)	237 (100%)	0.123
**Male**	226 (93%)	16 (7%)	242 (100%)	

**Table 4:** Age (in means and standard deviations) for each LP and LC response.

Not failed		Failed		*p*-value (ANOVA)
LP Mean	Standard Deviation	Mean	Standard Deviation	
3.65	1.76	3.16	1.64	0.006

Not failed		Failed		*p*-value (ANOVA)
LC Mean	Standard Deviation	Mean	Standard Deviation	
3.53	1.74	3.44	1.84	0.752

**Table 5:** Estimates of failure probability for LP for each hearing and age combination.

Hearing	Age	Probability
**Not failed**	**2**	28.7%
	**3**	24.2%
	**4**	20.3%
	**5**	16.9%
**Failed**	**2**	60.7%
	**3**	55.2%
	**4**	49.5%
	**5**	43.8%

Legend: A logistic regression model was used to test the effect of risk (probability) associated with each variable after adjusting for age.

**Table 6:** Estimates of the probability of LC deficits according to hearing screening results.

Hearing	Probability
**Not failed**	6.8%
**Failed**	19.4%

Legend: A logistic regression model was used to test the effect of risk (probability) associated with each variable.
